# Supplementary figures and images for: Genome-Wide Identification of the AGC Protein Kinase Gene Family Related to Photosynthesis in Rice (Oryza sativa)
Source: Int J Mol Sci. 2022 Oct 19;23(20):12557. doi: 10.3390/ijms232012557 (PMC9603967; doi:10.3390/ijms232012557)

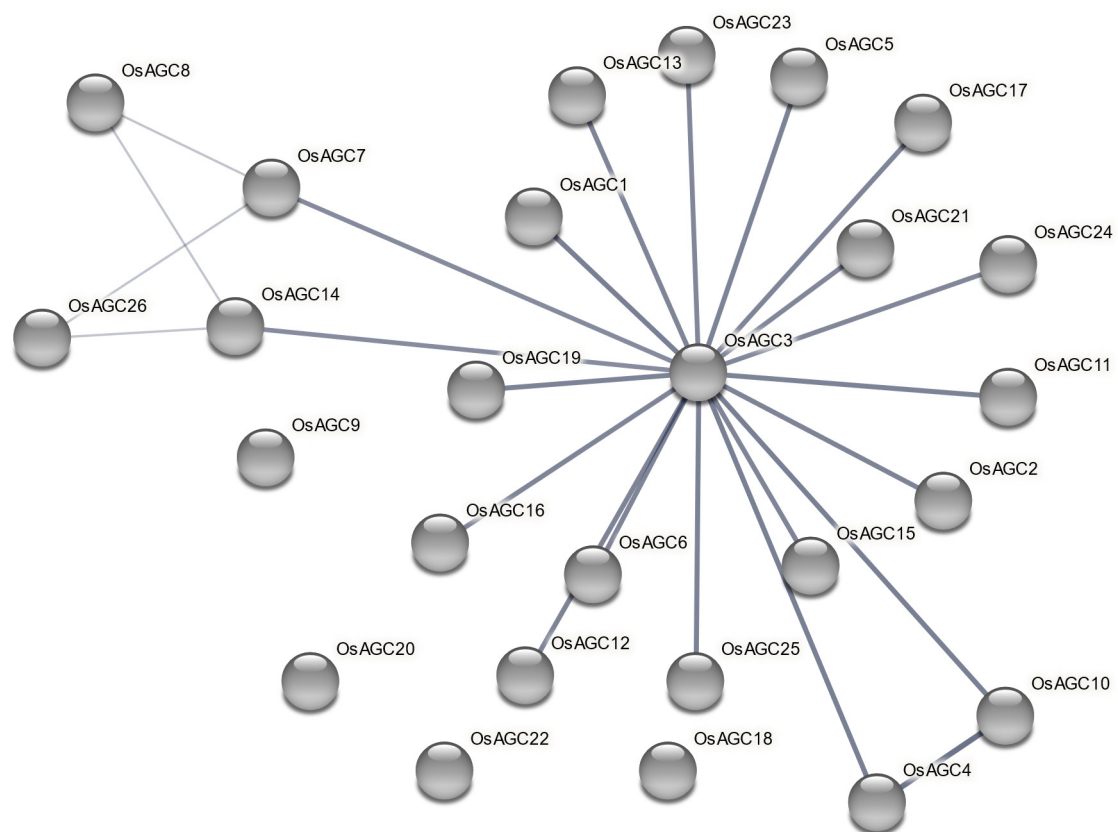

**Figure S1.** Protein-protein interaction network for OsAGC proteins in rice.

Supplement: Supplementary file 1 [file ijms-23-12557-s001.zip › Supplemental Figure S1.pdf]
